# An Altered Splicing Registry Explains the Differential ExSpeU1-Mediated Rescue of Splicing Mutations Causing Haemophilia A

**DOI:** 10.3389/fgene.2019.00974

**Published:** 2019-10-10

**Authors:** Dario Balestra, Iva Maestri, Alessio Branchini, Mattia Ferrarese, Francesco Bernardi, Mirko Pinotti

**Affiliations:** ^1^Department of Life Sciences and Biotechnology, University of Ferrara, Ferrara, Italy; ^2^Department of Experimental and Diagnostic Medicine, University of Ferrara, Ferrara, Italy

**Keywords:** RNA splicing, splicing mutations, human disease, ExSpeU1, Haemophilia A

## Abstract

The exon recognition and removal of introns (splicing) from pre-mRNA is a crucial step in the gene expression flow. The process is very complex and therefore susceptible to derangements. Not surprisingly, a significant and still underestimated proportion of disease-causing mutations affects splicing, with those occurring at the 5’ splice site (5’ss) being the most severe ones. This led to the development of a correction approach based on variants of the spliceosomal U1snRNA, which has been proven on splicing mutations in several cellular and mouse models of human disease. Since the alternative splicing mechanisms are strictly related to the sequence context of the exon, we challenged the U1snRNA-mediated strategy in the singular model of the exon 5 of coagulation factor (F)VIII gene (*F8*) in which the authentic 5’ss is surrounded by various cryptic 5’ss. This scenario is further complicated in the presence of nucleotide changes associated with FVIII deficiency (Haemophilia A), which weaken the authentic 5’ss and create/strengthen cryptic 5’ss. We focused on the splicing mutations (c.602-32A > G, c.602-10T > G, c.602G > A, c.655G > A, c.667G > A, c.669A > G, c.669A > T, c.670G > T, c.670+1G > T, c.670+1G > A, c.670+2T > G, c.670+5G > A, and c.670+6T > C) found in patients with severe to mild Haemophilia A. Minigenes expression studies demonstrated that all mutations occurring within the 5’ss, both intronic or exonic, lead to aberrant transcripts arising from the usage of two cryptic intronic 5’ss at positions c.670+64 and c.670+176. For most of them, the observed proportion of correct transcripts is in accordance with the coagulation phenotype of patients. In co-transfection experiments, we identified a U1snRNA variant targeting an intronic region downstream of the defective exon (Exon Specific U1snRNA, U1sh7) capable to re-direct usage of the proper 5’ss (∼80%) for several mutations. However, deep investigation of rescued transcripts from +1 and +2 variants revealed only the usage of adjacent cryptic 5’ss, leading to frameshifted transcript forms. These data demonstrate that a single ExSpeU1 can efficiently rescue different mutations in the *F8* exon 5, and provide the first evidence of the applicability of the U1snRNA-based approach to Haemophilia A.

## Introduction

In higher eukaryotes, the information necessary for protein synthesis is scattered across the gene, where the coding segments (exons) represent a minor proportion. Therefore, the exon recognition and the removal of the non-coding sequences (introns) from pre-mRNA are essential for proper gene expression, and this process (splicing) is carried out by a huge macromolecular complex named spliceosome. The first step of the spliceosome assembly involves binding of the small nuclear ribonucleoprotein U1 (U1snRNP) to the 5’ splice site (5’ss) by complementarity with the 5’ tail of its RNA component, U1snRNA (Roca et al., 2005). Not surprisingly, nucleotide changes occurring at the 5’ss, by interfering with its recognition and eventually leading to aberrant splicing events, are commonly associated with severe clinical phenotypes and are widely (9%) reported in human inherited diseases (http://www.hgmd.org/). This information led us to develop a correction strategy based on U1snRNAs variants designed to restore the complementarity with the mutated 5’ss (compensatory U1snRNA) (Pinotti et al., 2009). Unexpectedly, we also demonstrated the correction potential of engineered U1snRNAs targeting intronic sequences downstream of the defective exon (Exon Specific U1snRNAs; ExSpeU1), which are active on mutations occurring at the 5’ss, 3’ss as well as within exon (Alanis et al., 2012). The efficacy has been proven both in several cellular (Glaus et al., 2011; Schmid et al., 2011; Balestra et al., 2015; van der Woerd et al., 2015; Dal Mas et al., 2015a; Dal Mas et al., 2015b; Rogalska et al., 2016; Tajnik et al., 2016; Scalet et al., 2017; Scalet et al., 2018; Balestra et al., 2019; Balestra and Branchini, 2019; Scalet et al., 2019) and animal (Balestra et al., 2014; Balestra et al., 2016; Rogalska et al., 2016; Donadon et al., 2018b; Donadon et al., 2019; Lin et al., 2019) models of human disease.

However, the exon definition is very complex and, besides the splice sites, involves a series of splicing regulatory elements, which lead to the choice of the correct splice junctions and disfavor usage of the several cryptic splice sites (De Conti et al., 2013). Therefore, depending on the context, nucleotide changes can trigger different aberrant splicing mechanisms with which correction approaches, such us the U1snRNA-mediated one, must cope.

**Figure 1:**
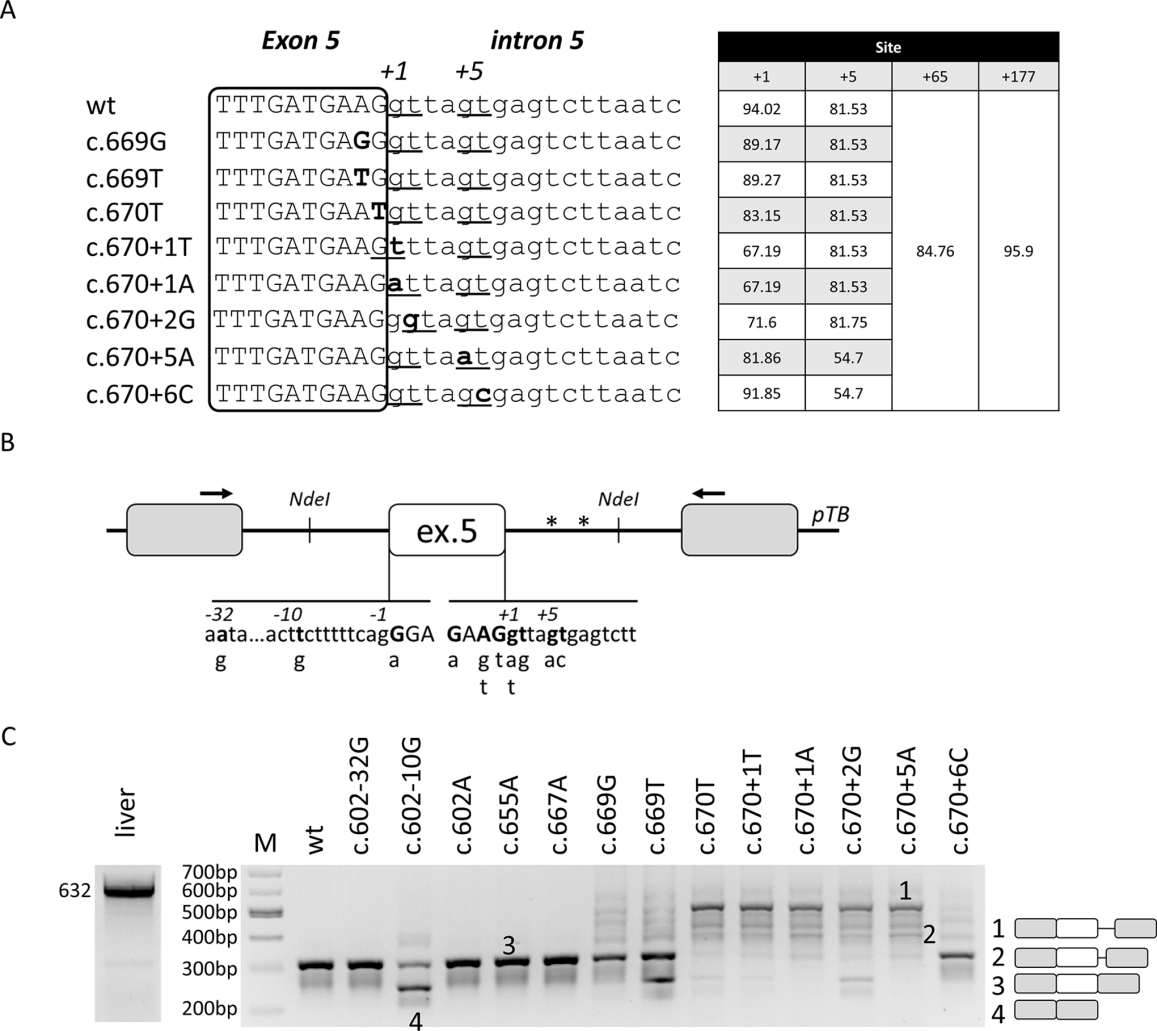
Nucleotide variants of *F8* exon 5 induce aberrant splicing, ranging from exon skipping to cryptic 5’ss usage.**(A)** Bioinformatic analysis of 5’ss in the wild-type context or upon introduction of nucleotide changes reported into the HA mutation database at www.factorviii-db.org/ and https://databases.lovd.nl/shared/genes/F8. Their score is based on HFR matrix according to the Human Splicing Finder online software (www.umd.be/HSF/). Sequences of exon (boxed) and intron 5 are indicated respectively in upper and lower cases. Nucleotide changes are indicated in bold and the predicted 5’ss are underlined, with the relative scores reported on the right. **(B)** Schematic representation of the *F8* exon 5 minigene cloned into the pTB vector. Exonic and intronic sequences are represented by boxes and lines, in upper and lower cases, respectively. Nucleotides reported in HA patients, together with their relative nucleotide changes, are indicated in bold and in the lower part of the figure. Asterisks represent cryptic 5’ss located at position +65 and +177 in intron 5. **(C)** Evaluation of *F8* alternative splicing patterns in HEK293T cells transiently transfected with minigene variants. The schematic representation of the transcripts (with exons not in scale) is reported on the right. Numbers represent respectively the transcripts with +176 (1) and +64 (2) intronic nucleotides, wild-type transcripts (3), or those missing exon 5 (4). Amplified products were separated on 2% agarose gel. M, 100 bp molecular weight marker. Amplification of mRNA spanning exon 4 through exon 8 in human liver cDNA is reported on the left.

Here, we challenged the ExSpeU1s in the *F8* exon 5 as a model of context in which the authentic 5’ss is surrounded by various cryptic 5’ss (**Figure 1A**), a scenario further complicated by the occurrence of nucleotide changes at the 5’ss that are associated with coagulation factor VIII (FVIII) deficiency (Haemophilia A, HA) (Bolton-Maggs and Pasi, 2003). Minigene expression studies indicated that these changes alter the delicate interplay among 5’ss, which can be apparently re-balanced by a ExSpeU1 targeting a downstream intronic sequence. However, the deep investigation of the rescued transcripts from +1 and +2 variants revealed that the ExSpeU1 re-directed the usage of the newly-created cryptic 5’ss, thus vanishing the correction attempt. These data demonstrate the applicability of the ExSpeU1 to HA-causing mutations and strengthen the importance of the sequence context in dictating the splicing outcome.

## Materials and Methods

### Creation of Expression Vectors

To create the pF8wt plasmid, the genomic region of the human *F8* gene (NC_000023.11) spanning from c.602-464 to c.670+773 was amplified from genomic DNA of a normal subject with primers i4F-i5R using high-fidelity Pfu DNA-polymerase (Transgenomic, Glasgow, UK). The *F8* amplicon was sequentially cloned into the pTB expression vector (gift of prof. F. Pagani, ICGEB, Trieste, Italy) by exploiting the *Nde*I restriction site.

To create the pF8-32A > G, pF8-10T > G, pF8.602G > A, pF8.655G > A, pF8.667G > A, pF8.669A > G, pF8.669A > T, pF8.670G > T, pF8+1G > T, pF8+1G > A, pF8+2T > G, pF8+5G > A, and pF8+6T > C plasmids, the nucleotide changes were introduced into the pF8wt minigene by site-directed mutagenesis (QuickChange II Site-Directed Mutagenesis Kit, Stratagene, La Jolla, CA, USA).

The pU1F8d, pU1F8s7, pU1F8s16, pU1F8s25 expression vectors for the modified U1snRNAs were created by replacing the sequence between the *Bgl*II and *Xba*I restriction sites with a PCR generated with a U1-specific forward primer (containing the modified 5’ tail of the U1snRNA) and a reverse primer base-pairing downstream the *Xba*I cloning site.

The pU7a,b,c,d expression vectors for the modified U7snRNAs were created as previously reported (Balestra et al., 2015). Briefly, a PCR containing the modified binding site of the engineered U7snRNA has been generated by using the primers indicated in **Supplementary Table 1** and cloned into the pSP64 plasmid (gift from Franco Pagani, ICGEB, ITA) after digestion with *Stu*I and *Xba*I restriction sites.

All vectors have been validated by sequencing.

Sequences of oligonucleotides are provided in **Supplementary Table 1**.

### Expression in Mammalian Cells and mRNA Studies

Human Embrionic Kidney 293T (HEK293T) cells were cultured as previously described (Farrarese et al., 2018). Cells were seeded on twelve-well plates and transfected with the Lipofectamine 2000 reagent (Life Technologies, Carlsbad, CA, USA), according to the manufacturer’s protocol.

Five hundred nanograms of pF8 minigene variants were transfected alone or with a molar excess (1.5X) of the pU1/pU7 plasmids. Total RNA was isolated 24 h post-transfection with Trizol (Life Technologies), reverse-transcribed with random primers and amplified using the Pfu DNA-polymerase (Transgenomic, Glasgow, UK) with primers Alfa and Bra. The same DNA polymerase and primers 4F and 8R were used to evaluate the *F8* splicing patterns in human liver. Densitometric analysis for the quantification of correct and aberrant transcripts was performed using the ImageJ software (https://imagej.net). 

For denaturing capillary electrophoresis analysis, the amplified fragments were labeled by using primers Alfa and the fluorescently-labeled (FAM dye) Bra and run on an ABI-3100 instrument (Waltham, MA, USA).

Three independent experiment were conducted for each variant and condition.

### Computational Analysis

Computational prediction of splice sites and of splicing regulatory elements was conducted by using the Human Splicing Finder (www.umd.be/HSF/) online software.

## Results

### The Computational Analysis Predicts Several Competing 5’ss

The bioinformatic analysis (www.umd.be/HSF3/index.html) predicts that *F8* exon 5 is well defined, as demonstrated by the high scores of the 5’ss and 3’ss (94,02 and 94,62, respectively) (**Figure 1A**). Moreover, it predicts three cryptic 5’ss in intron 5, two of them located at nucleotide positions +65 and +177 bp, and one in the proximity (+5) of the authentic one. All of them have a score (81, 85, and 96 for +5, +65, and +177 cryptic 5’ss, respectively) close to that of the authentic 5’ss and, based on the HSF matrix, above the threshold of 80.

In this model, we chose to analyze a panel of nucleotide variants occurring at the 5’ss (c.667G > A, c.669A > G, c.669A > T, c.670G > T, c.670+1G > T, c.670+1G > A, c.670+2T > G, c.670+5G > A, c.670+6T) or 3’ss (c.602-32A > G, c.602-10T > G, c.602G > A), and associated with different degree of HA severity (**Table 1**). The introduction of nucleotide changes at the 5’ss is predicted to weaken the authentic 5’ss, with changes at the highly conserved positions +1 and +2 being the most detrimental ones (**Figure 1A**). Interestingly, due to the genomic context of *F8* exon 5, the introduction of the c.670+1G > T and c.670+2T > G variants is predicted to insert new and shifted alternative 5’ss that, if used, would lead to aberrant transcripts differing for only one nucleotide in size, as compared to the correct one (-1 for c.670+1G > T and +1 for c.670+2T > G).

**Table 1:** Features of the nucleotide changes reported in the *F8* mutation databases (www.factorviii-db.org/ and https://databases.lovd.nl/shared/genes/F8) investigated in the study. The HA coagulation phenotype is defined on the basis of FVIII cofactor activity levels (severe <1%; moderate, 1-5%; mild 5-40%).

Location in gene	No of Patients reported	Mutation	Amino acid change	Coagulation phenotype
Intron 4	1	c.602-32A > G		Severe
Intron 4	1	c.602-10T > G		Mild
Exon 5	1	c.602G > A	p.Gly201Glu	Severe
Exon 5	15	c.655G > A	p.Ala219Thr	Mild
Exon 5	1	c.667G > A	p.Glu223Lys	Severe
Exon 5	1	c.669A > G	p.Glu223Glu	Mild
Exon 5	2	c.669A > T	p.Glu223Asp	Severe
Exon 5	2	c.670G > T	p.Gly224Trp	Moderate
Intron 5	3	c.670+1G > T		Severe
Intron 5	1	c.670+1G > A		Severe
Intron 5	1	c.670+2T > G		Severe
Intron 5	3	c.670+5G > A		Severe
Intron 5	4	c.670+6T > C		Mild/Moderate

This provided us with an informative model to assess 5’ss competition and aberrant splicing mechanisms as well as the suitability of the ExSpeU1 as a correction approach.

### The *F8* Exon 5 Nucleotide Changes Lead to Aberrant Splicing, Ranging From Exon Skipping to Usage of Cryptic Intronic 5’ss

To investigate splicing mechanisms in the *F8* exon 5 context, we created a *F8* minigene including the *F8* exon 5 and the surrounding introns (**Figure 1B**). Expression of the wild type (wt) minigene in HEK293T cells indicated that exon 5 is well defined, and this pattern recapitulates the complete inclusion observed in human liver mRNA (**Figure 1C**), thus validating our experimental approach.

Since even exonic nucleotide changes such as missense mutations might affect the splicing code, we screened for the presence of exonic splicing enhancers (ESEs), which were predicted by computational analysis. To this purpose we exploited antisense U7snRNA variants designed to target and mask the candidate ESEs (U7a,b,c) or partially the authentic 5’ss (U7d). Co-expression of these U7snRNA variants with the wt minigene revealed that only the control U7d affected splicing, and partially induced exon 5 skipping (**Supplementary Figure 1**). Since these data did not provide elements for a selection among the many exonic nucleotide variations annotated in the HA databases (www.factorviii-db.org/ and https://databases.lovd.nl/shared/genes/F8), we only investigated the c.655G > A change, being the most frequent missense change in *F8* exon 5.

Expression studies with the minigene variants and the analysis of splicing patterns by RT-PCR and conventional electrophoresis demonstrated that some changes (c.602-32A > G, c.602G > A, c.655G > A, and c.667G > A) were ineffective on splicing (**Figure 1C**). Differently, the c.602-10T > G, c.669A > T, c.670G > T, c.670+1G > T, and c.670+2T > G variants led, to various extent, to exon 5 skipping. Moreover, all mutations within the 5’ss (c.669A > T, c.669A > G, c.670G > T, c.670+1G > T, c.670+1G > A, c.670+1G > T, c.670+2T > G, c.670+5G > A, and c.670+6T) led to alternative transcripts originating from the usage of cryptic 5’ss located 65 and 177 bp downstream of the authentic 5’ss (**Figure 1C**). All aberrant transcripts, confirmed by sequencing (**Supplementary Figure 2**), account for a deleted and frame-shifted mRNA, with two premature stop codons at intronic positions c.673-678.

Taken together these data identified mutations causing aberrant splicing and provided candidates to explore splicing correction by ExSpeU1s.

### A Unique ExSpeU1 Is Able to Rescue Multiple Mutations but Not Variants at +1 and +2 Positions Due to an Altered Splicing Registry

**Figure 2:**
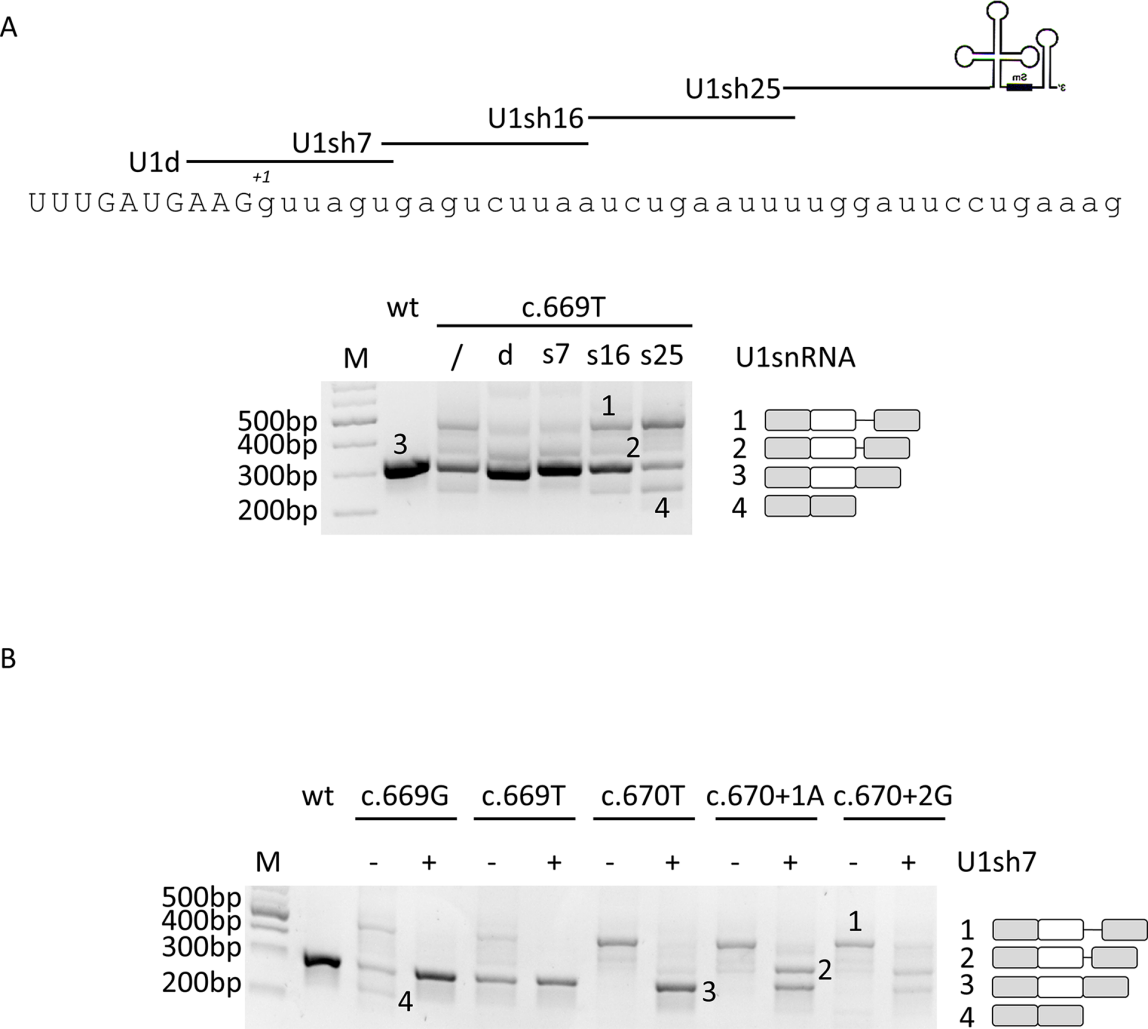
The ExSpeU1 can rescue multiple mutations and, apparently, also those at position +1 and +2 of 5’ss. **(A)** Evaluation of *F8* alternative splicing patterns in HEK293T cells transiently transfected with the wild-type or c.669T minigenes alone or in combination with a 1.5X molar excess of engineered U1-expression plasmids. The sequence of 5’ss of *F8* exon 5 and of binding sites of engineered U1snRNAs are represented in the upper part of the figure. The schematic representation of the transcripts (with exons not in scale) is reported on the right. **(B)** *F8* alternative splicing patterns in HEK293T cells transfected with mutant minigenes alone or in combination with the ExSpeU1sRNAs7. The schematic representation of the transcripts is reported on the right. Amplified products were separated on 2% agarose gel. M, 100 bp molecular weight marker.

In the attempt to restore proper exon definition, we designed a compensatory U1snRNA and three ExSpeU1 with perfect complementarity to the wild-type 5’ss or the adjacent intronic sequences, respectively (**Figure 2A**). The efficacy of these U1snRNA variants has been initially evaluated on the c.669A > T change since mutations at -2 position of the 5’ss have been previously shown to be rescuable by the modified U1snRNA-based approach (Alanis et al., 2012). Co-transfection experiments led us to select the compensatory U1sRNA (U1d) and one ExSpeU1 (U1sh7) that appreciably rescued splicing. In particular, the densitometric analysis of bands revealed that co-expression of the U1d and U1sh7 was associated with an increase of correctly spliced transcripts (from 52 ± 3% to 71 ± 3% or 75 ± 4% for U1d and U1sh7, respectively) (**Figure 2A**).

Based on these results and on the fact that the ExSpeU1, by binding to a less conserved intronic region, potentially ensures higher exon specificity (Rogalska et al., 2016; Donadon et al., 2019), the U1sh7 was selected for further investigation on an enlarged panel of variants. Co-transfection experiments showed that the U1sh7 remarkably rescued the c.669A > G, c.669A > T, and c.670G > T variants (> 80% of correct transcripts), and also appeared to have a correction effect (from 0% to ∼40%) on those at the conserved +1 (c.670+1A) and +2 (c.670+2G) positions (**Figure 2B**).

**Figure 3:**
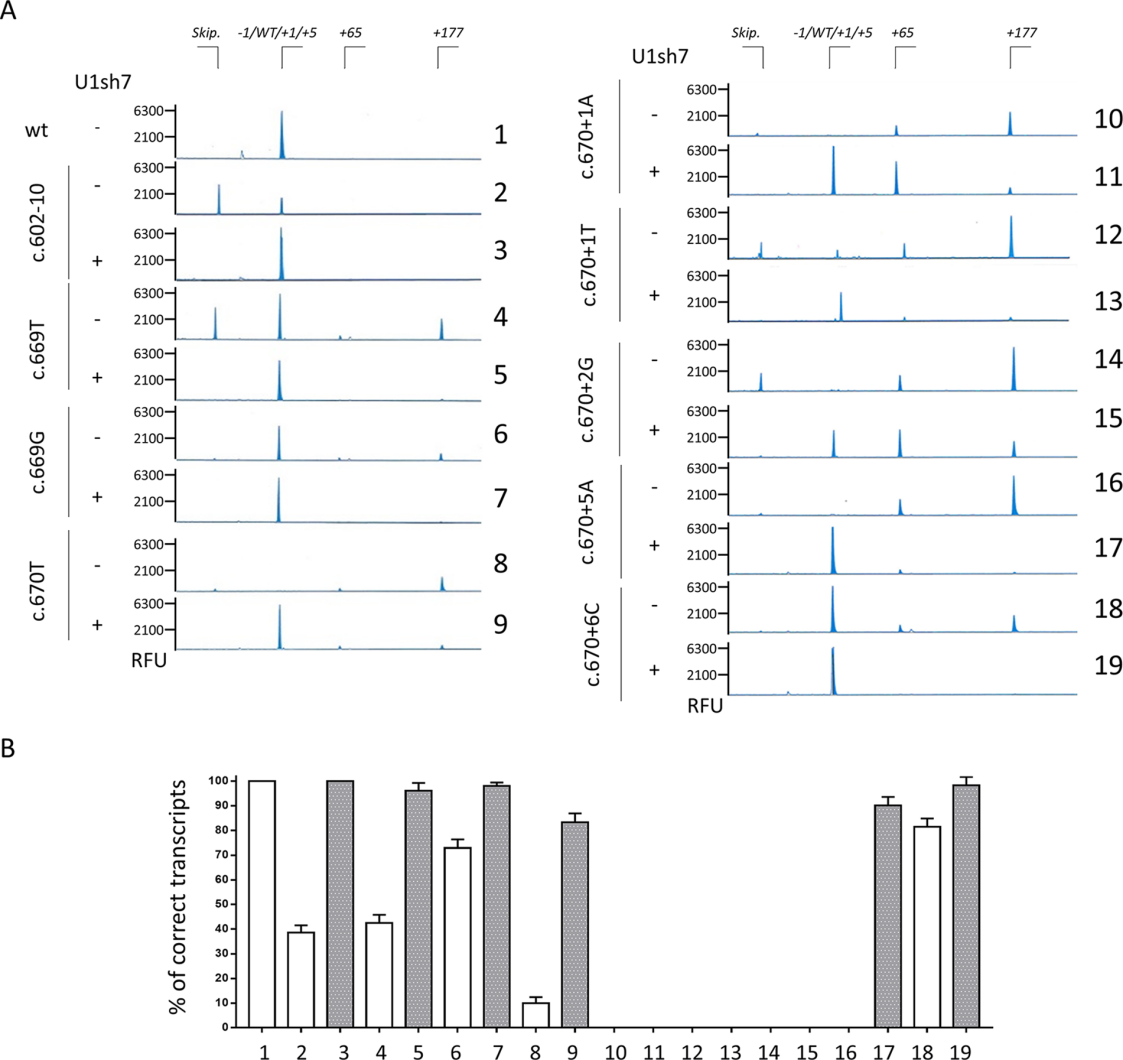
Capillary electrophoresis-mediated analysis of splicing reveals the usage of an altered splicing registry.**(A)** Analysis of splicing patterns in HEK293T cells expressing mutant minigenes alone or in combination with ExSpeU1sRNAs7 by denaturing capillary electrophoresis of fluorescently-labelled PCR products. The amount of transcripts is represented by the area under each peak. The scheme of transcripts is reported on top. RFU: Relative Fluorescence Units. **(B)** Relative amount of correctly spliced transcripts in HEK293T cells transfected as in panel A and analyzed by denaturing capillary electrophoresis. The white and grey histograms report the percentage of correct transcripts expressed as mean ± SD from three independent experiments before or after treatment with U1sh7.

The unexpected appearance of transcripts with a size compatible with correct splicing even for mutants at positions +1 and +2 prompted us to further analyze the splicing outcome by fluorescent labeling of amplicons followed by denaturing capillary electrophoresis (**Figure 3** and **Supplementary Figure 3**). This approach, in cells expressing the +1 and +2 variants alone, revealed the presence of trace levels of aberrant transcripts differing for only a few nucleotides (-1, +1 and +4 bp). In particular, the -1 and +1 aberrant transcripts were identified only in the c.670+1T and c.670+2G context (7.1 ± 2.4% and 1.3 ± 0.7%, respectively), while the +4 transcript was detected for the c.670+1T (1.1 ± 0.8%), c.670+2G (1.4 ± 0.8%), and c.669T mutant (1 ± 0.6%).

Through this approach, we then evaluated the effect of the U1sh7, which re-directed the spliceosome on the exon-intron junction, as indicated by the generally decreased transcripts arising from the usage of the distal cryptic 5’ss. However, due to the mutations at the crucial positions, the U1sh7 forced the usage of the adjacent cryptic 5’ss, created/strengthened by mutations, and remarkably increased the proportion of +4 forms for the c.670+1T (from 1.1 ± 0.8 to 72.6 ± 2.2%) and c.670+1A (from 0 to 55.3 ± 2.1%) and +1 forms for the c.670+2G (from ∼1% to ∼34%) variants.

Concerning the other mutants, the denaturing capillary electrophoresis permitted us to complete the proper evaluation of the U1sh7-mediated rescue on the selected panel of mutations. As shown in **Figure 3B**, the co-expression of the U1sh7 led to an appreciably increased proportion of correct transcripts for the c.602-10T > G variant at the 3’ss and the c.669A > T, c.669A > G, c.670G > T, c.670+5G > A, and c.670+6T variants at the 5’ss.

Taken together, our data dissected further the aberrant splicing patterns associated with HA-causing mutations and identified a unique ExSpeU1 able to rescue multiple mutations, except for +1/+2 variants suffering from an unfavorable context.

## Discussion

The advent of next-generation sequencing has enormously expanded the number of gene variations associated with human diseases, thus posing the problem of identifying the causative ones. This is particularly difficult for nucleotide changes that, being at the exon-intron boundaries or within introns, are candidate to affect splicing since their precise effect is hardly predictable by computational tools. The scenario is further complicated by the overlapped splicing and amino acid codes within exons, which might lead to missense changes exerting their pathogenic roles by altering the splicing process rather than the protein biology (Tajnik et al., 2016; Donadon et al., 2018a).

In this context, the experimental evaluation of the impact of nucleotide changes on splicing is mandatory to help diagnosis and counseling. Here, we addressed this issue in a singular gene context, namely the *F8* exon 5, where various exonic changes and multiple cryptic 5’ss are respectively located within or in the proximity of the authentic 5’ss, thus complicating the selection of the right one.

The analysis of the splicing pattern of the c.602-10A > G nucleotide change, with appreciable levels (∼38%) of correctly spliced transcripts, is consistent with the mild coagulation phenotype reported in the patient. Conversely, the c.602G > A (p.G201E), c.655G > A (p.A219T), and c.667G > A (p.E223G) missense variants are not associated with significant splicing alterations, indicating that FVIII deficiency is mainly caused by the underlying amino acid substitutions impairing protein biosynthesis/function. This observation is also strengthened by the data with antisense U7snRNAs that do not support the presence of important regulatory exonic elements in the *F8* exon 5, which might have been altered by exonic changes. However, in the proximity of the 5’ss, the splicing code overlaps with the amino acid registry, with the splicing one being the first used in the gene expression flow. Concerning mutations occurring within the 5’ss, it is worth noting that the exonic c.669A > G (p.E223E), c.669A > T (p.E223D), and c.670G > T (p.G224W) variants clearly alter splicing. Whereas the c.669A > T is mainly associated with exon skipping and loss of exon definition, the c.669A > G and particularly the c.670G > T variants lead to partial intron retention, with the usage of a strong intronic 5’ss at position +177. Noticeably, splicing analysis of variants at different positions of the same triplet (c.667G > A, c.669A > G, and c.669A > T) coding for glutamic acid at position 233 in FVIII resulted in different splicing outcomes, ranging from exon skipping to the usage of cryptic splice 5’ss or null. This finding highlights the need for careful evaluation of the effects of exonic changes on splicing, due to the limited help of bioinformatic analysis in predicting the pathogenicity of nucleotide changes. It is worth noting that levels of correct transcripts for the c.669A > T (p.E223D) (∼40%) and c.670G > T (∼10%) variants suggest that the associated FVIII deficiency (moderate/severe) would arise from a combination of splicing and protein impairment. On the other hand, the mutations at the intronic positions +1/+2/+5 were not compatible with correct splicing. Differently, the +6 variant led to remarkable levels of correctly spliced transcripts, in accordance with the severe or mild coagulation phenotypes reported in HA patients, respectively.

The knowledge of aberrant splicing patterns lays the foundation for the exploration of correction approaches for therapeutic purposes, as we did in several other human disease models. Intervention at the mRNA level has the advantage of maintaining the physiological gene regulation and is based on delivery of small coding cassettes, thus allowing the exploitation of any viral vector strategy. Among the different strategies, engineered U1sRNAs demonstrated the ability of rescuing multiple mutation types, including changes at 5’ss, 3’ss as well as within exons, in cellular and animal models of human disease (Glaus et al., 2011; Schmid et al., 2011; Balestra et al., 2014; Balestra et al., 2015; van der Woerd et al., 2015; Dal Mas et al., 2015a; Dal Mas et al., 2015b; Balestra et al., 2016; Rogalska et al., 2016; Tajnik et al., 2016; Scalet et al., 2017; Scalet et al., 2018; Donadon et al., 2018b; Donadon et al., 2019; Scalet et al., 2019). Noticeably, modified U1snRNAs were shown to preserve their correction effect even when targeting intronic regions downstream of the defective exon (ExSpeU1) through a mechanism that, unlike antisense oligonucleotides blocking an intronic element, involves U1snRNP assembly, spliceosome activation, and recruitment of splicing factors (Martínez-Pizarro et al., 2018; Rogalska et al., 2016). Moreover, this second generation of modified U1snRNAs potentially ensures higher exon and gene specificity since their base-pairing ability with intronic, and thus less conserved, sequences, as supported by recent studies (Donadon et al., 2019; Rogalska et al., 2016). Notwithstanding, the off-target effect of each ExSpeU1 has to be carefully assessed when approaching clinics.

In the *F8* exon 5 context, we identified an ExSpeU1 able to restore proper exon definition in the presence of multiple mutations, located at both 3’ss or 5’ss. Interestingly, we observed that variants at + 1 and + 2 position of 5’ss, not thought to be rescuable by modified U1snRNA due to of their high degree of conservation, resulted in transcripts with size comparable with that of correctly spliced ones. Recently, the T > C transition at position +2 of 5’ss has been demonstrated to be rescuable by modified U1snRNA (Scalet et al., 2019), a finding compatible with the observation that a small fraction of introns removed by U2-type spliceosome has cytidine at position +2 (Burset et al., 2000). There are also examples of mutations at +1 position that are compatible with correct processing (Hartmann et al., 2010), which potentially open the possibility to rescue them. Here, to dissect the elusive nature of transcripts, we exploited the denaturing capillary electrophoresis, a strategy able to distinguish amplicons differing by only one nucleotide. The analysis of the splicing patterns revealed the usage of cryptic 5’ss created by mutations (c.670+1G > T and c.670+2T > G) leading to transcripts respectively shorter or longer of one single nucleotide. Unfortunately, due to the altered registry of the 5’ss, the ExSpeU1 further promoted the usage of the 5’ss other than the authentic one, thus vanishing the correction effect.

In conclusion, through molecular characterization of various *F8* exon 5 variants occurring at the 3’ss or 5’ss, or within the exon, we demonstrated for the first time the ability of a unique ExSpeU1 to rescue multiple HA-causing *F8* mutations. Moreover, our findings highlight the need to investigate the effect on splicing of nucleotide changes, particularly of those occurring in exonic sequences, and suggest a careful inspection of the sequence context and evaluation of transcripts to avoid over-interpretations, with implications for diagnosis and counseling.

## Data Availability Statement

All datasets generated for this study are included in the manuscript/**Supplementary Files**.

## Ethics Statement

Ethics approval for this study was not required as per the local legislation. Notwithstanding, the DNA sample from control patient was used after obtaining the informed consent.

## Author Contributions

All authors contributed significantly to the manuscript. The manuscript was conceived and prepared by DB, MP, and FB. IM performed the capillary electrophoresis analysis and AB, MF, and DB performed the experiments. Overall, manuscript clarity was reviewed by all authors, and all approved its content.

## Funding

Authors would like to acknowledge the support provided by the Early Career Bayer Haemophilia Awards Program (BHAP 2017, DB).

## Conflict of Interest

MP is inventor of a patent (PCT/IB2011/054573) on modified U1snRNAs.

The remaining authors declare that the research was conducted in the absence of any commercial or financial relationships that could be construed as a potential conflict of interest.
