# Supplementary figures and images for: An Altered Splicing Registry Explains the Differential ExSpeU1-Mediated Rescue of Splicing Mutations Causing Haemophilia A
Source: Front Genet. 2019 Oct 10;10:974. doi: 10.3389/fgene.2019.00974 (PMC6796300; doi:10.3389/fgene.2019.00974)

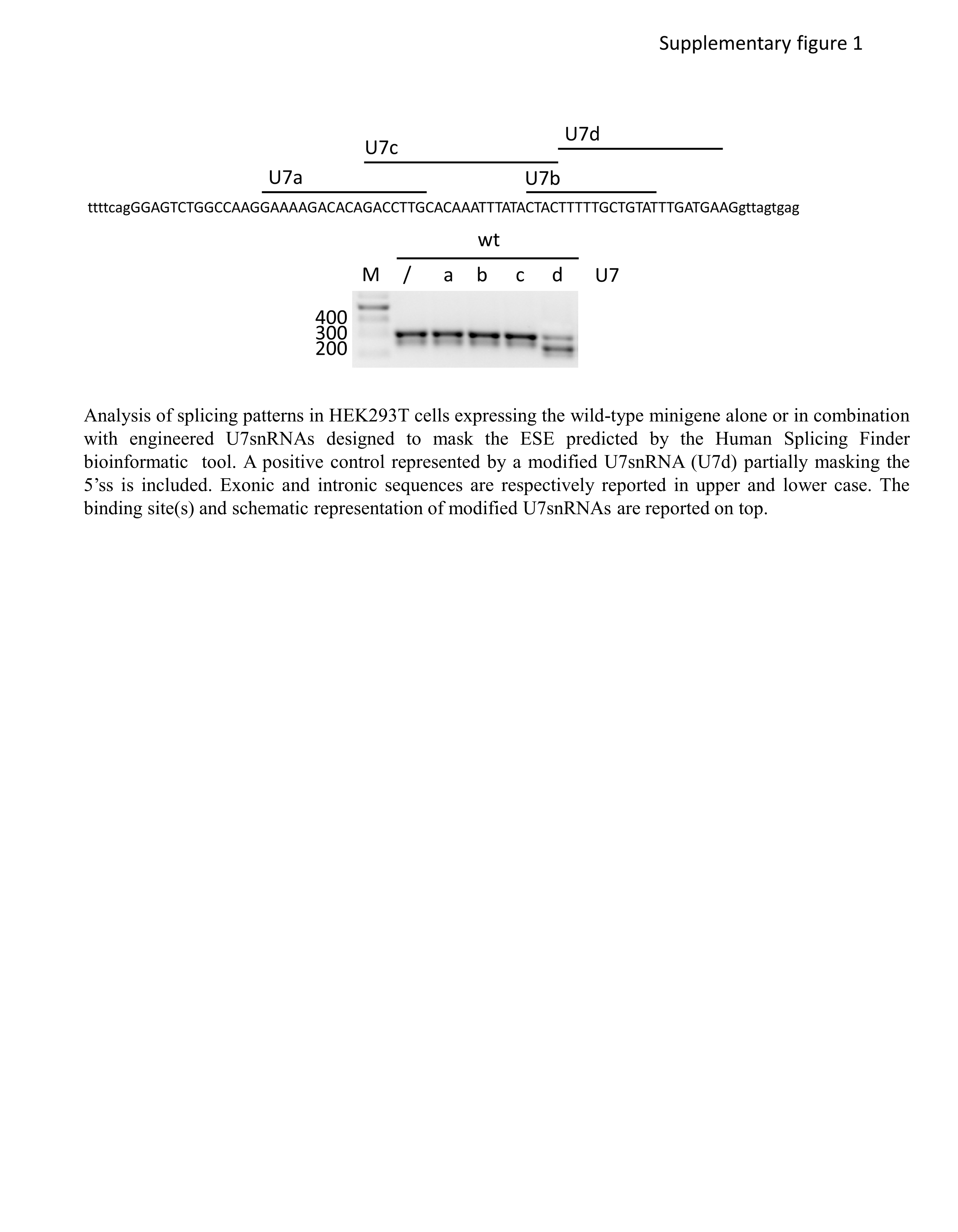

Supplement: Supplementary file 1 [file Image_1.tif]

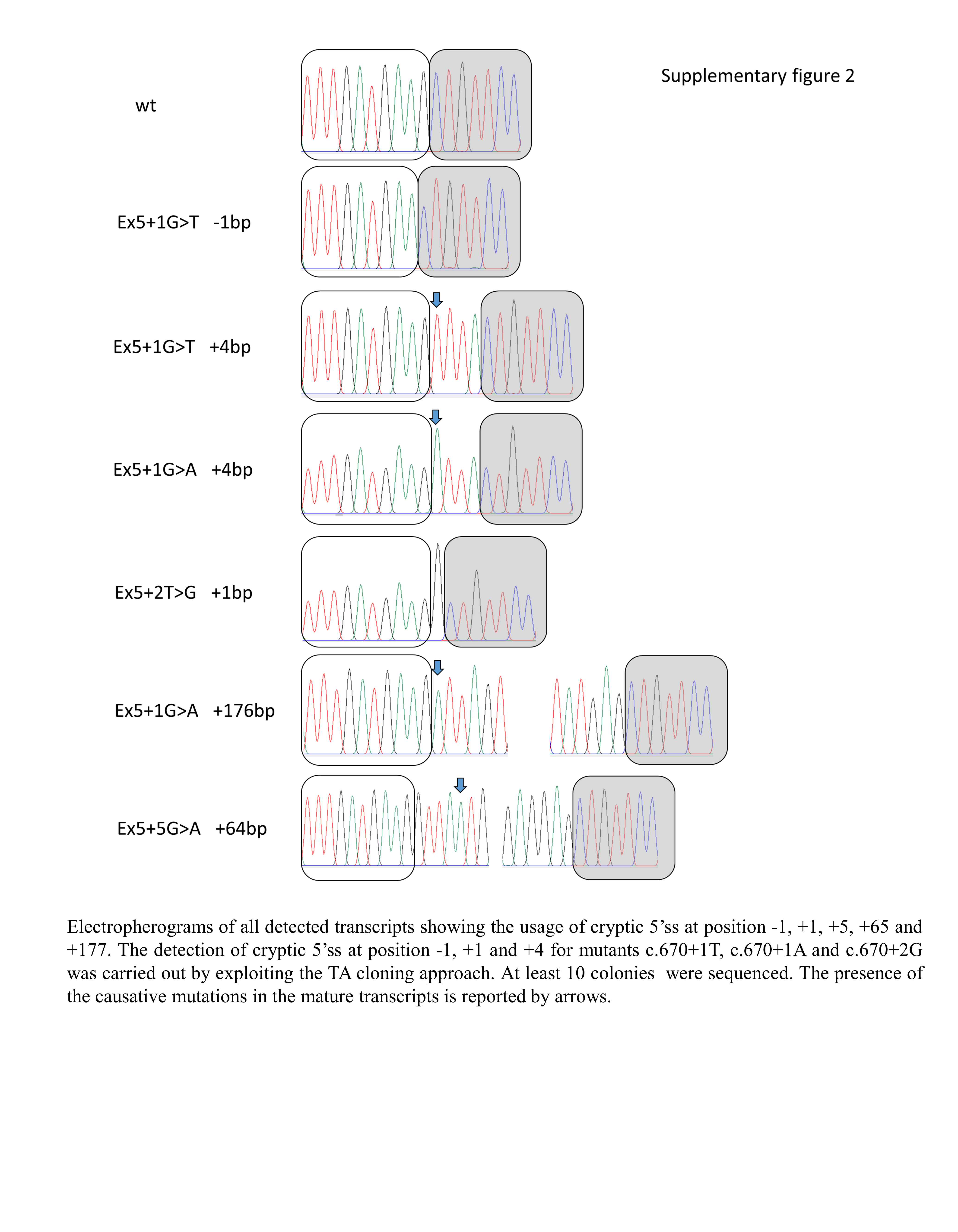

Supplement: Supplementary file 2 [file Image_2.tif]

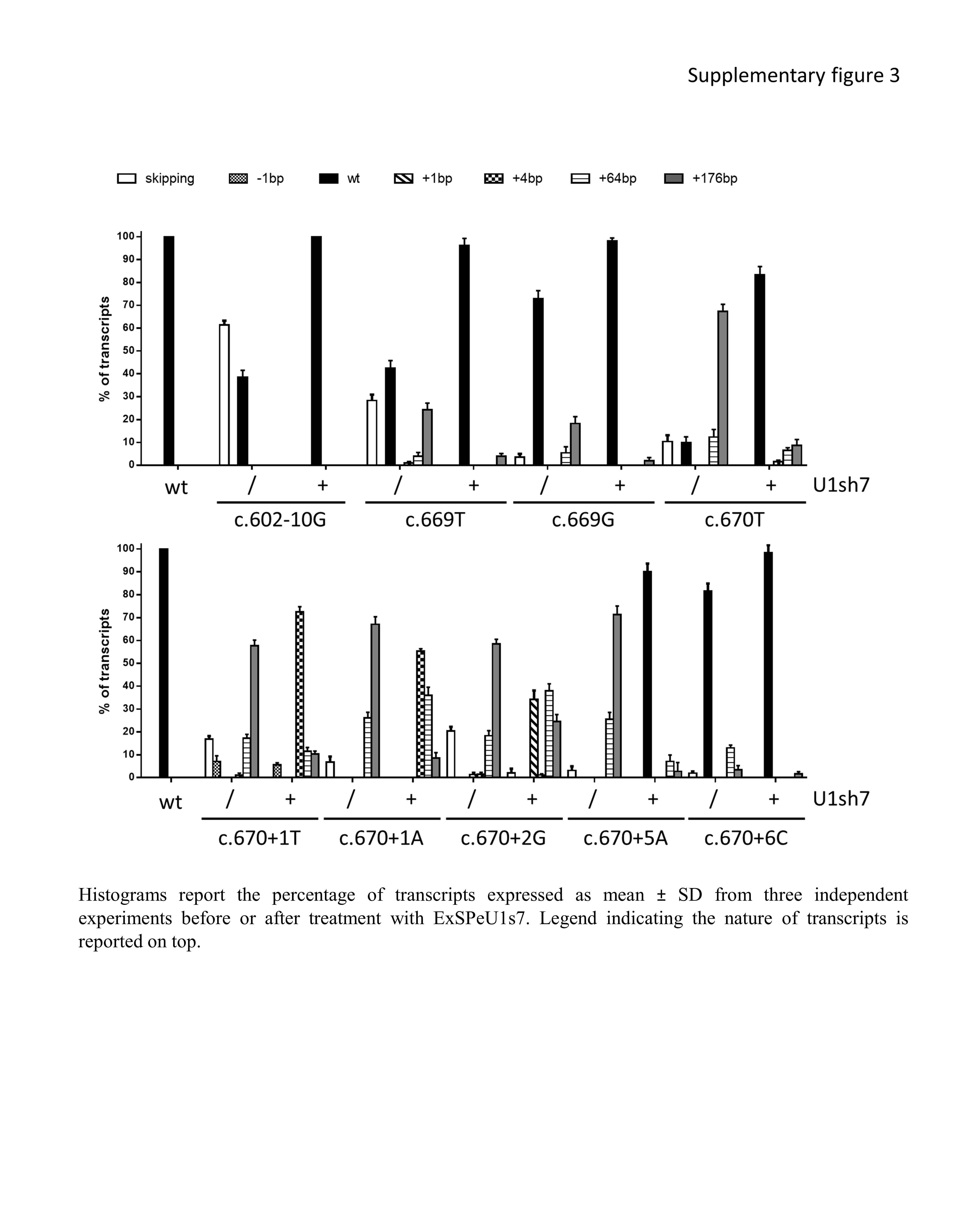

Supplement: Supplementary file 3 [file Image_3.tif]
