# Supplementary material for: An Altered Splicing Registry Explains the Differential ExSpeU1-Mediated Rescue of Splicing Mutations Causing Haemophilia A
Source: Front Genet. 2019 Oct 10;10:974. doi: 10.3389/fgene.2019.00974 (PMC6796300; doi:10.3389/fgene.2019.00974)
Supplement: Supplementary file 4 [file Table_1.docx]

| Name | | Sequence 5’🡪3’ |
| --- | --- | --- |
| To create minigenes and for mutagenesis | | |
| i4F | forward | aaacatatgtgtgagtataattagaagcactgtgg |
| i5R | reverse | aaacatatgattcttttgtagagatgtggtttcg |
| ex5 -32G | forward | gagattttttaagtagaagataaatg |
| ex5 -32G | reverse | catttatcttctacttaaaaaatctc |
| ex5 -10G | forward | gataaatgttctcactgctttttcagggagtc |
| ex5 -10G | reverse | gactccctgaaaaagcagtgagaacatttatc |
| ex5 602A | forward | cttctttttcagagagtctggccaag |
| ex5 602A | reverse | cttggccagactctctgaaaaagaag |
| ex5 655A | forward | tatactactttttactgtatttgatg |
| ex5 655A | reverse | catcaaatacagtaaaaagtagtata |
| ex5 667A | forward | gctgtatttgataaaggttagtgag |
| ex5 667A | reverse | ctcactaacctttatcaaatacagc |
| ex5 669G/T | forward | ctgtatttgatgakggttagtgagtc |
| ex5 669G/T | reverse | gactcactaaccmtcatcaaatacag |
| ex5 670T | forward | ctgtatttgatgaatgttagtgagtc |
| ex5 670T | reverse | gactcactaacattcatcaaatacag |
| ex5 670+1T/A | forward | ctgtatttgatgaagwttagtgagtcttaatc |
| ex5 670+1T/A | reverse | gattaagactcactaawcttcatcaaatacag |
| ex5 670+2G | forward | gctgtatttgatgaagggtagtgagtcttaatctg |
| ex5 670+2G | reverse | cagattaagactcactacccttcatcaaatacagc |
| ex5 670+5A | forward | gctgtatttgatgaaggttaatgagtcttaatctgaattttg |
| ex5 670+5A | reverse | caaaattcagattaagactcattaaccttcatcaaatacagc |
| ex5 670+6C | forward | gatgaaggttagcgagtcttaatctg |
| ex5 670+6C | reverse | cagattaagactcgctaaccttcatc |
| To create pU7p variants | | |
| U7aF | forward | acagaggcctttccgcacaaggtctgtgtcttttcaatttttggag |
| U7bF | forward | acagaggcctttccgcaatacagcaaaaagtagaatttttggag |
| U7cF | forward | acagaggcctttccgcagtagtataaatttgtgcaaggtcaatttttggag |
| U7dF | forward | acagaggcctttccgcattcatcaaatacagcaaaaaaatttttggag |
| SP6 | reverse | atttaggtgacactatag |
| To create pU1 variants | | |
| U1F8ex5d | forward | aggcccaagatctcatctcactaacgcaggggagataccatgatca |
| U1F8ex5s7 | forward | aggcccaagatctcatttaagactcgcaggggagataccatgatca |
| U1F8ex5s16 | forward | aggcccaagatctcataaattcagagcaggggagataccatgatca |
| U1F8ex5s25 | forward | aggcccaagatctcattggattcctgcaggggagataccatgatca |
| U1cR | reverse | atagaatacaagcttgcatgcctg |
| To evaluate splicing patterns | | |
| 4F | forward | tttctcatgtggacctggtaaaaga |
| 8R | reverse | gataaaggaaggagagttgtcatca |
| Alfa | forward | caacttcaagctcctaagccactgc |
| Bra | reverse | taggatccggtcaccaggaagttggttaaatca |

**Supplementary Table S1**

List of primers
